# Measurement and analysis of prevertebral soft tissue width of cervicothoracic region using magnetic resonance images

**DOI:** 10.12669/pjms.301.3706

**Published:** 2014

**Authors:** Lin Wang

**Affiliations:** Lin Wang, Department of Neurosurgery, Tianjin 4th Centre Hospital, Tianjin, 300140, China.

## Abstract

***Objective***: To evaluate the characterization of normal prevertebral soft tissue width of cervicothoracic region using magnetic resonance images.

***Methods***: Prevertebral soft tissue width in the cervicothoracic region of 165 patients was measured using magnetic resonance images (MRI). The mean and standard deviation of these measurements were calculated and Spearman's correlation analysis was conducted to study the influences of age, sex and vertebral level on prevertebral soft tissue width.

***Results***: The prevertebral soft tissue width in 165 subjects were measured and documented, and the value at each level is significantly different from those at adjacent level (p<0.05). In addition, the width of prevertebral soft tissue in female and younger is significantly smaller than those in male and elders (p>0.05).

***Conclusion***: Vertebral level, sex and age have significant influence on prevertebral soft tissue width.

## INTRODUCTION

The cervicothoracic region is a complex, unstable anatomic site at which the mobile cervical spine joins the rigid thoracic spine, causing the region susceptible to injury.^1^ The injury such as the disruption of blood vessels covering the anterior aspects of the spine^2^ and some non-bony conditions may cause the prevertebral soft tissue width increasing. The increased prevertebral soft tissue width has been regarded as a marker of cervical spine trauma.^3^^,^^4^ In addition, the cervicothoracic region is the site where many abnormalities including vascular anomalies, esophageal lesions and emphysema may occur^5^, which also result in the increase of prevertebral soft tissue width. As a result, a better understanding of the morphology of prevertebral soft tissue is helpful in accurate diagnosis and etiology analysis in cervicothoracic region.

Some authors measured and analyzed the prevertebral soft tissue width in cervical region using X-radiographs^6^^,^^7^, but few authors performed a study in cervicothoracic region. Mullin and colleagues^3^ measured and analyzed the prevertebral soft tissue width in cervicothoracic region using X-radiographs, while the inherent deficiency of X-radiographs may not allow good visualization in the region due to the overlying osseous and soft tissue structures of the shoulder and upper chest. In contrast MRI provides the greatest range of information and accurate delineation of soft tissue and osseous structures, enabling detection of subtle abnormalities with great sensitivity.^8^^,^^9^ In the study of Stemper and colleagues^10^, prevertebral soft tissue width was measured in cervical region from C2 to C7 using MRI, and the analysis of characteristics of prevertebral soft tissue width was performed. While, his study focused on the cervical instead of cervicothoracic region. Up to now, the MRI characteristics of prevertebral soft tissue width in cervicothoracic region is still unclear.

Therefore, we performed an evaluation of characteristics of prevertebral soft tissue width in cervicothoracic region using MRI and the objective was to: (1) Provide level-by-level normative data from healthy individuals; (2) Study the correlation between prevertebral soft tissue width and age, gender and vertebral level to facilitate the spine surgeons to better analyze the pathological changes in the region.

## METHODS

In the current study, one hundred sixty-five subjects’ sagittal magnetic resonance images were reviewed. All MRI were obtained in subjects who presented through the Emergency Department of the authors’ hospital. Subjects presented with complaints of neck pain, neck stiffness, headache, and shoulder pain resulting from head or neck trauma. All MRI selected for measurement and analysis were deemed to be those without evidence of trauma or pathology, regardless of patient symptoms.^11^

Patient charts were subsequently reviewed for history of cervical, thoracic, cardiopulmonary or gastroesophageal pathology or prior surgical intervention that might compromise the integrity of our data. Subjects who previously underwent endotracheal intubation, nasogastric tube placement, or tracheostomy or patients with radiographic evidence of prior surgery were excluded from the study.^3^ The current study was approved by the ethical committee of authors’s hospital.

The measurement of prevertebral soft tissue width for each level from C6 to T4 was performed on the mid-sagittal reformatted images in DICOM format using Image J software. The mean and standard deviation were calculated and documented. The width of prevertebral soft tissue was determined as the minimum distance from the craniocaudal midpoint of the each vertebral body anterior cortex from C6 to T4 to the posterior aspect of the air column.^10^

Analysis of variance was used to determine the significant difference in prevertebral soft tissue width, and spearman's correlation analysis was carried out to study the influences of gender, age and vertebral level on prevertebral soft tissue width. The statistics was performed using SPSS 17.0 (SPSS, Inc., Chicago, IL) and the level of significance was set at p<0.05.

## RESULTS

One hundred sixty-five subjects’ sagittal magnetic resonance images were reviewed in the current study. Among 165 subjects, ninety-eight were male and 67 were female. The mean age was 48.5 years old and the age ranged from 18 to 78 years old.

A summary of prevertebral soft tissue width in 165 subjects is presented in Table-I. A trend of progressive decrease in the means is found from C6 to T2, then a progressive increase from T2 to T4, T2 has the lowest value and is significantly lower than those at other levels (p<0.05). In addition, the value at each level is significantly different from those at adjacent level (p<0.05). Spearman's correlation analysis reveals vertebral level (p<0.05) and age (p<0.05) has significant influence on prevertebral soft tissue width (p<0.05). In addition, the width of prevertebral soft tissue in female is significantly smaller than those in male (p>0.05).

Our measurement protocol revealed a satisfactory repeatability with intra- and interobserver correlations of 0.92 and 0.87.

## DISCUSSION

In the present study, we performed a measurement and analysis of prevertebral soft tissue width of cervicothoracic region in healthy individuals using magnetic resonance images. To the best of our knowledge, few studies have been published on the issues, and we believe the current study may facilitate surgeons to better analyze the pathological changes in the region.

The soft tissue thickness between the bony cervical vertebral column and the air space of the pharynx and trachea is constituted by the anterior longitudinal ligament, the longus capitis and longus coli musculature, and the prevertebral and alar fascia. Many authors have suggested the measurement of prevertebral soft tissue width on a lateral cervical radiograph to be a useful indicator of cervical spine injury.^3^ Some authors performed measurements of prevertebral soft tissue width using cervical X-radiographs. In the Penning’s study, the mean soft tissue width at C6 level was 15.1mm and at the C7 level is 13.9mm; James^7^ reported a mean width of 16.23mm to C6 and 15.31mm to C7. These results are relatively larger than those in the current study and we attribute it to the magnification effect of X-radiographs.

**Table-I:** The measurement results of prevertebral soft tissue width (mm) in all subjects

*Level*	*Male*	*Female*	*ALL*
C6	15.21±0.71	14.11±0.98	14.61±0.89
C7	14.13±0.45	13.28±0.31	13.58±0.37
T1	12.97±0.41	10.89±0.39	11.75±0.38
T2	10.33±0.49	9.21±0.50	9.95±0.45
T3	11.41±0.65	10.77±0.58	11.09±0.59
T4	13.23±0.67	12.65±0.59	12.98±0.60

Some authors studied the effect of gender, age and vertebral level on prevertebral soft tissue width in cervical region. In the analysis of cervical X-radiographs of normal adults, Oon found no significant sex difference in the soft tissue width.^13^ However, in the study of 150 patients using cervical X-radiographs, Chi^6^ found the prevertebral soft tissue width was statistically thicker in male patients; Mullin^3^ also found the mean prevertebral soft tissue width for female subjects was smaller than for male subjects at C7 and T1 levels. In the current study, we also found the comparison of prevertebral soft tissue width between males and females had significant difference, and the values in male patients are larger. Some author^3^suggested the difference in prevertebral soft tissue width between sexes was attributed to the larger body habitus in males and the effect of magnification from the greater distance between spinal column and radiographic cassette instead of an intrinsic difference in prevertebral soft tissue thickness between the sexes. However, in the current study, our measurements were performed using MRI which overcome the above shortcomings of X-radiographs. Consequently, the lager value of prevertebral soft tissue width in male subjects can be confirmed in the current study.

Sistrom^14^ found patients older than 70 years had smaller prevertebral soft tissue width. While, in the study from Mullin, comparison of the group of mean prevertebral soft tissue width revealed a marked increase in the groups that included patients 41 years and older. In the present study, we also found the similar results and the elderly patients have relatively larger soft tissue width than younger ones. In our opinion, with the increasing age, the loss in disc height and vertebral body height may increase, together with the increased anterior osteophyte and local kyphosis, which may result in an increasing of prevertebral soft tissue width.

Moreover, the spearman's correlation analysis in the present study reveals that vertebral level has significant influence on the prevertebral soft tissue width. A progressive decrease in the mean was found from C6 to T2, then a progressive increase from T2 to T4, T2 has the lowest value. Also, the mean of prevertebral soft tissue width at each level is significantly different from those at adjacent levels, demonstrating that the verterbral level has significant influence on the width of the prevertebral soft tissue. The studies from stemper^10^ and Mullin^3^ came up with the same conclusions. The anatomic differences in the soft tissues and thoracic organs anterior to the spinal column at different levels may explain the current results.

## CONCLUSION

In the current study, we provided a normative data of prevertebral soft tissue width in cervicothoracic region of healthy individuals. We found the age, gender and vertebral level have significant influence on the preverterbral soft tissue width in cervicothoracic region. However, some factors, such as the degree of lung inflation and motion of trachea in breath, may also have influence on the width, but weren’t studied. Subsequently, more studies need to be performed in the future.
